# Efficacy and mechanisms of *Gynostemma pentaphyllum* as a medicine food homology herb in glycemic control: a meta-analysis with review

**DOI:** 10.3389/fphar.2026.1731723

**Published:** 2026-04-15

**Authors:** Ruoshi Wang, Yuqing Wang, Yanfei Mo, Heng Zhang, Gaoxiang Ma

**Affiliations:** 1 Department of Cardiology, Pukou Hospital of Chinese Medicine Affiliated to China Pharmaceutical University, School of Traditional Chinese Pharmacy, China Pharmaceutical University, Nanjing, China; 2 Science and Education Section, Pukou Hospital of Chinese Medicine Affiliated to China Pharmaceutical University, School of Traditional Chinese Pharmacy, China Pharmaceutical University, Nanjing, China; 3 Department of Hematology and Oncology, Children’s Hospital of Nanjing Medical University, Nanjing, China

**Keywords:** diabetes, fasting plasma glucose, glycemic control mechanisms, *Gynostemma pentaphyllum*, meta-analysis

## Abstract

**Introduction:**

*Gynostemma pentaphyllum* (Thunb.) Makino is a traditional “medicine food homology” plant widely consumed in Asia to promote health. Its safety profile makes it a promising diabetes candidate. This study systematically evaluates its efficacy in glycemic control and elucidates its mechanisms of action.

**Methods:**

We conducted a meta-analysis of clinical studies from PubMed, CNKI, and Embase. Random-effects models calculated pooled mean differences (MD) for fasting plasma glucose (FPG), 2-h postprandial glucose (2hPG), and glycated hemoglobin (HbA1c). A systematic review also summarized its antidiabetic mechanisms.

**Results:**

Eight studies involving 584 patients were included. *Gynostemma pentaphyllum* significantly reduced FPG (MD = −0.79 mmol/L, 95% CI = −1.08 to −0.51), HbA1c (MD = −1.01%, 95% CI = −1.41 to −0.61), and 2hPG (MD = −0.90 mmol/L, 95% CI = −1.95 to −0.15). Long-term and monotherapy treatments showed superior efficacy. The mechanistic review revealed hypoglycemic effects via multiple pathways, including enhanced glucose uptake, reduced glucose production, increased insulin secretion, and improved insulin resistance.

**Discussion:**

Combined clinical and mechanistic findings demonstrate that *Gynostemma pentaphyllum* provides effective glycemic control through multi-target pharmacological mechanisms, solidifying its potential as a comprehensive therapeutic approach for diabetes management.

## 1 Introduction

*Gynostemma pentaphyllum* (Thunb.) Makino [Cucurbitaceae; *Gynostemmatis Pentaphylli Herba*] is a classic example of a medicine food homology herb, widely recognized in traditional Chinese medicine and dietary practices across East and Southeast Asia (Hu et al., 2009; Nguyen et al., 2021). In China, historical records of the plant date back to the Ming Dynasty. The classical text *Jiuhuang Bencao* (1406 CE) described it as a wild edible herb used during famine periods, while the *Compendium of Materia Medica* documented its medicinal applications for conditions such as hematuria, throat swelling, tumors, and traumatic injuries (Liang et al., 2023). According to traditional Chinese medicine theory, *Gynostemma pentaphyllum* is characterized by a slightly bitter taste and neutral properties and is traditionally believed to clear heat, detoxify the body, tonify qi, and promote general vitality (Chen et al., 2022).

Beyond China, *Gynostemma pentaphyllum* has also been incorporated into the traditional practices of several Asian countries. In Japan, the plant is commonly known as “Amachazuru” or “Ganchaman,” and it has long been consumed as a herbal tea and health beverage due to its mild sweetness and perceived tonic effects. In Korea and other parts of Southeast Asia, including Vietnam, Thailand, and Laos, the plant is traditionally used as both a medicinal herb and a leafy vegetable, reflecting its dual role as food and medicine (Nguyen et al., 2021). In many rural communities across these regions, the leaves and young stems are prepared as teas, soups, or simple dishes, making the plant widely regarded as a natural health-promoting herb associated with longevity and metabolic balance (Niu et al., 2013).

Modern phytochemical studies have identified a wide range of bioactive constituents in *Gynostemma pentaphyllum*, particularly saponins (gypenosides), flavonoids, and polysaccharides, which contribute to its diverse pharmacological activities (Lou et al., 2021). The long-standing ethnopharmacological use of this plant, together with its favorable safety profile as a dietary herb, has attracted increasing scientific interest in its potential therapeutic applications. In particular, accumulating experimental and clinical evidence suggests that *Gynostemma pentaphyllum* may exert beneficial effects on metabolic disorders, especially diabetes mellitus, by regulating glucose metabolism and improving metabolic homeostasis (Chen et al., 2023).

Diabetes, particularly Type 2 diabetes (T2D), has become a major global health concern. The pathophysiology of diabetes is complex, involving deficiencies in insulin secretion, insulin resistance, and excessive hepatic glucose production. Modern therapeutic strategies have shifted from relying solely on glucose-lowering medications to a more comprehensive approach that addresses multiple metabolic pathways, including reducing glucose production, increasing glucose uptake, and improving insulin secretion and sensitivity (Fujita et al., 2010; Asche et al., 2011; DeFronzo et al., 2015; Kyle et al., 2016; Davila and McCormack, 2024).

Reducing hepatic glucose production is key in diabetes management. Metformin lowers fasting blood glucose by inhibiting hepatic glucose production (Fujita et al., 2010), and SGLT2 inhibitors like dapagliflozin also modulate this process while promoting renal glucose excretion (Cuypers et al., 2013; Vivian, 2015). Targeting liver metabolism can improve both glucose control and cardiovascular health. Increasing glucose uptake and utilization is a crucial strategy in diabetes management (Huang et al., 2023; Thupakula et al., 2024). Meanwhile, improving insulin sensitivity in peripheral tissues like muscle and fat can lower postprandial blood glucose (Hirshman and Horton, 1990; Bastard et al., 1998). In addition, improving insulin secretion and sensitivity is essential for controlling Type 2 diabetes, where insulin resistance limits glucose clearance (Arcidiacono et al., 2014; Yang et al., 2017). Enhancing insulin secretion or improving insulin sensitivity both contribute to better glycemic control. Moreover, increased insulin sensitivity further amplifies the hormone’s effectiveness in peripheral tissues (Tam and Sweeney, 2023).

Despite its extensive traditional use and the promising preclinical evidence supporting its hypoglycemic effects, the clinical translation of *Gynostemma pentaphyllum* remains challenging. While numerous randomized controlled trials (RCTs) have been conducted to evaluate its efficacy, the results often appear inconsistent due to variations in study populations, treatment durations, and the phytochemical composition of the botanical drug preparations used (Li et al., 2025). Furthermore, existing individual studies often lack sufficient power to provide a definitive quantitative assessment of its impact on core glycemic indicators such as FPG, 2hPG, and HbA1c (Begzati et al., 2025).

A high-quality systematic evaluation is critically needed to reconcile the observed variability in therapeutic outcomes. Moreover, the macroscopic clinical benefits observed in human trials have not been systematically correlated with the microscopic multi-target mechanisms of its specialized metabolites. Therefore, this study aims to conduct a rigorous meta-analysis of RCTs to quantitatively assess the efficacy of *Gynostemma pentaphyllum* in glycemic control. By integrating these clinical findings with a comprehensive review of its pharmacological pathways, we seek to provide a robust, evidence-based translational perspective on this “medicine food homology” species for modern diabetes management.

## 2 Meta-analysis of *Gynostemma pentaphyllum* for glycemic control

### 2.1 Search strategy and selection criteria

We aimed to identify published clinical trials assessing the efficacy of *Gynostemma pentaphyllum* (Jiaogulan) in patients with diabetes. Both extract and whole-plant forms of *Gynostemma pentaphyllum* were included in the search. We further restricted the search to trials with a primary outcome focusing on glycemic control (e.g., fasting blood glucose, HbA1c).

We searched the following databases: PubMed, CNKI (China National Knowledge Infrastructure), and Embase for reports published up to 1 October 2025, using the following search terms: “Gynostemma pentaphyllum”, “Jiaogulan”, “diabetes”, “blood glucose control”, “glycated hemoglobin”, “clinical trial”. The inclusion criteria for the studies were as follows: (1) The study population consisted of patients with type 2 diabetes; (2) The study design was a randomized controlled trial (RCT); (3) The intervention involved *Gynostemma pentaphyllum*, either as a standalone treatment or in combination with other medications; (4) The outcomes measured included fasting blood glucose, glycated hemoglobin (HbA1c), or other relevant blood glucose control indicators, with data available for pre- and post-treatment levels. The exclusion criteria for the studies were as follows: (1) unrelated to blood glucose control; (2) reusing published data; (3) involving non-diabetic patients; (4) non-clinical studies or non-RCTs; (5) control group without review articles, patents, case reports, or conference abstracts.

Data extraction was performed by two independent authors, with any discrepancies over study inclusion or data interpretation resolved by consensus.

### 2.2 Risk of bias assessment

The potential for publication bias was visually assessed using funnel plots. Asymmetry of the funnel plot could indicate potential publication bias, where smaller studies with less significant results were less likely to be published. A symmetric funnel plot suggested a low risk of publication bias (Supplementary Figure S1).

Sensitivity analysis was conducted to evaluate the robustness and reliability of the results. This analysis aimed to determine how sensitive the overall conclusions of the meta-analysis were to changes in the included studies, methodologies, or statistical assumptions (Supplementary Figure S2).

### 2.3 Data analysis

The primary aim of this meta-analysis was to evaluate the effects of *Gynostemma pentaphyllum* on glycemic control in patients with diabetes. The key outcomes assessed included: Fasting Plasma Glucose (FPG), 2-h Postprandial Glucose (2hPG), and Glycated Hemoglobin (HbA1c). For each of these outcomes, the summary statistics from the individual trials were extracted, as individual-level data were not available. Relevant treatment effects were calculated based on published trial reports, using mean differences (MD) with 95% confidence intervals (CIs) for continuous outcomes (FPG, 2hPG, HbA1c). The effect sizes from each study were combined using inverse variance-weighted averages of logarithmic treatment effects within random-effects models.

Subgroup analyses were conducted to investigate potential sources of heterogeneity and to explore how different variables may impact the treatment effects of *Gynostemma pentaphyllum* on glycemic control.

### 2.4 Results

**FIGURE 1:**
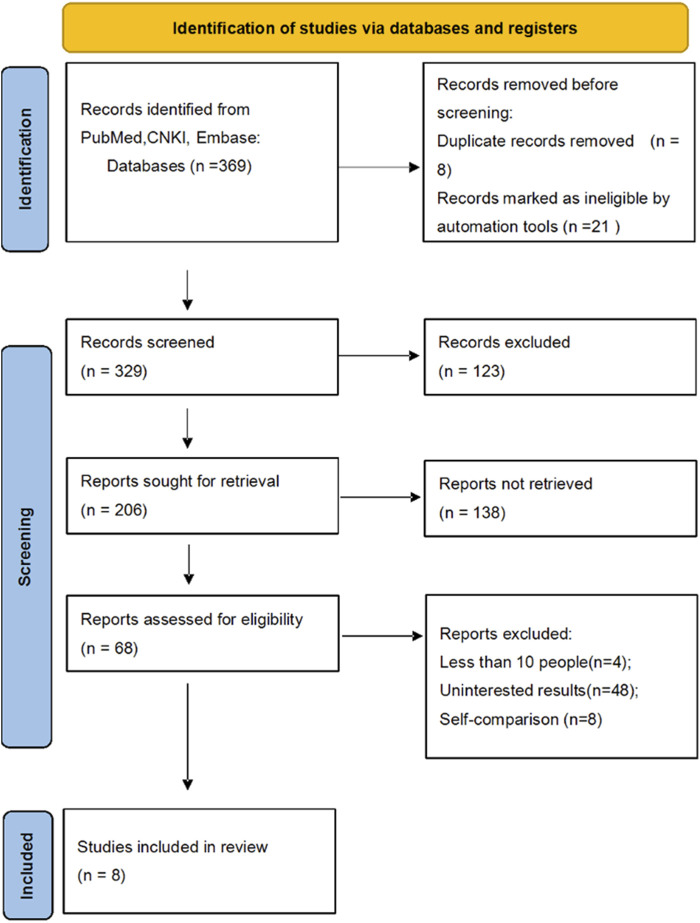
Flow diagram for the process of study selection. The flowchart illustrates the detailed study selection process: 369 records were initially identified, 29 were removed before screening, and 329 were screened. Subsequently, 123 were excluded, 206 were sought for retrieval, and 138 were not retrieved. Among the 68 assessed for eligibility, 60 were excluded for various reasons, yielding 8 studies for inclusion in the final review.

The process of identifying and including studies for the meta-analysis was illustrated in the flowchart. Initially, 369 records were identified from databases including PubMed, CNKI, and Embase. Before screening, 8 duplicate records and 21 records marked as ineligible by automation tools were removed. This left 329 records for screening, of which 123 were excluded. From the remaining 206 reports sought for retrieval, 138 could not be retrieved. Of the 68 reports assessed for eligibility, 48 were excluded due to irrelevant results and 4 due to having fewer than 10 participants (Table 1). Ultimately, 8 studies were included in the review (Figure 1).

**TABLE 1:** Clinical study summary of medications for diabetes management.

Study	Area	Sample size		Age		Treat		Dose		Duration	Outcome
		Experimental	Control	Experimental	Control	Experimental	Control	Experimental	Control		
Zhang et al. (2015)	China	32	32	47.9 ± 8.4	48.0 ± 8.5	GP	Yishenfu capsule	One dose/day, decoction of 210 mL, taken in three parts	288mg/dose/day	180 days	FPG
Zhang et al. (2015)	China	32	32	47.9 ± 8.4	48.1 ± 8.3	GP	Standard treatment	One dose/day, decoction of 210 mL, taken in three parts	Standard treatment	180 days	FPG
Yu (2017)	China	8	8	45 ± 5		Acetaminophen capsule + GP saponin	Simvastatin tablet	Acetaminophen (1 capsule/dose) + GP (1 tablet/dose), 3 times/day	20 mg/day	90 days	FPG
Shi and Fang (2016)	China	89	89	52.05 ± 7.93	51.54 ± 8.12	Metformin HCl tablet + atorvastatin Calcium + GP	Metformin HCl tablet + atorvastatin calcium	0.5 g, 3 times/day, 20 mg, 1 time/day	0.5 g, 3 times/day, 20 mg, 1 time/day	56 days	FPG, 2hPG, HbA1c
Ma et al. (2017)	China	50	50	56.64 ± 8.50	56.50 ± 8.92	GP + Xuezhi Kang + Metformin	Metformin	GP 5 g, soaked twice; Xuezhikang0.6g,2 times/day; Metformin 0.25 g, 3 times/day	0.25 g, 3 times/day	144 days	FPG, 2hPG, HOMA-IR
Chen Huichao, 2023	China	28	28	54.77 ± 9.46	54.83 ± 9.87	Repaglinide + GP total saponins tablets	Repaglinide	1 mg, 3 times/day 1 tablet, three times/day	1 mg, 3 times/day	60 days	FPG, 2hPG, HbA1c
Huyen et al. (2010)	Vietnam	11	12	63.5 ± 6.5	57.2 ± 8.2	GP extract tea	Green tea	3 g/packet, twice/day, powdered, infused in hot water	3 g/packet, twice/day, powdered, infused in hot water	72 days	HOMA-I, HOMA-β, FPG
Huyen (2012)	Vietnam	12	13	55.0 ± 9.0		GP extract tea	Green tea	3 g/packet, twice/day, powdered, infused in hot water	3 g/packet, twice/day powdered, infused in hot water	72 days	FPG, HbA1c, HOMA-IR
Wang et al (1998)	China	20	20	41.0 ± 12.9		Diet control + GP tea	Diet control + glibenclamide	GP 10 g, three times/day	2.5 mg, three times/day	14 days	FPG
Wang et al (1998)	China	20	20	41.0 ± 12.9		Diet control + GP tea + glibenclamide	Diet control + glibenclamide	GP 10 g, three times/day 2.5 mg, three times/day	2.5 mg, three times/day	14 days	FPG

* Indicates different groups within the same experiment.

We summarized the characteristics and outcomes of 8 studies conducted between 1998 and 2023, involving participants from China and Vietnam. The studies focused on various treatments and their effects on different health outcomes, such as fasting plasma glucose (FPG), and 2-h postprandial glucose (2hPG). The treatments included traditional Chinese medicine, Western medicine, and dietary interventions. The duration of the studies ranged from 14 to 180 days. The outcomes measured included FPG, 2hPG, HbA1c, and homeostasis model assessment of insulin resistance (HOMA-IR) (Wang et al., 1998; Huyen et al., 2010; 2012; Zhang et al., 2015; Shi and Fang, 2016; Yu, 2017; Shi, 2020; Chen et al., 2023).

**FIGURE 2:**
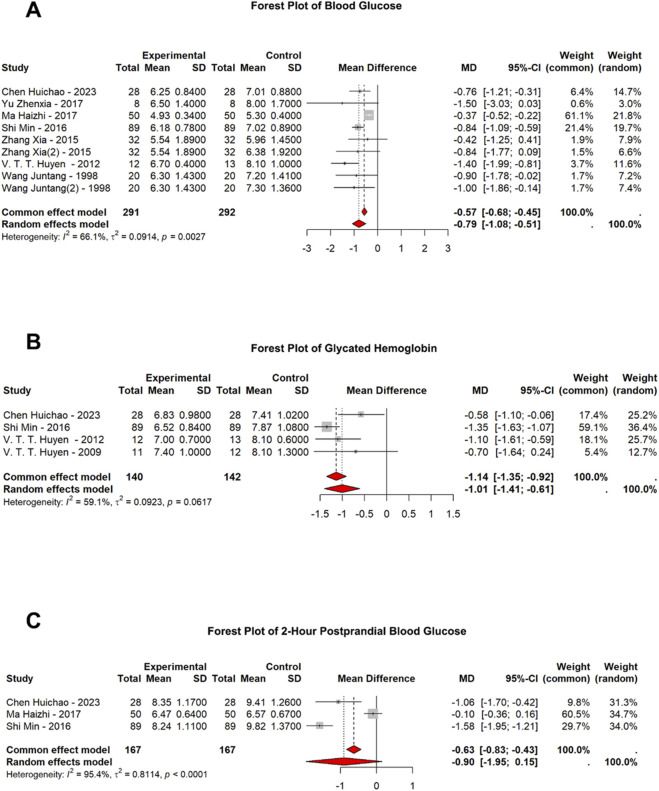
Efficacy of Gynostemma pentaphyllum in Managing Diabetes Mellitus. **(A)** Pooled Mean Effect Sizes of Gynostemma pentaphyllum on Blood Glucose Levels: Based on 8 studies. Calculated using a random-effects model. Overall mean differences, -0.79 mmol/L; 95% CI, -1.08 to -0.51. **(B)** Pooled Mean Effect Sizes of Gynostemma pentaphyllum on Glycated Hemoglobin (HbA1c) Levels: Based on 4 studies. Calculated using a random-effects model. Overall mean differences, -1.01%; 95% CI, -1.41 to -0.61. **(C)** Pooled Mean Effect Sizes of Gynostemma pentaphyllum on 2-Hour Postprandial Glucose (2hPG) Levels: Based on 3 studies. Calculated using a random-effects model. Overall mean differences, -0.90 mmol/L; 95% CI, -1.95 to -0.15; I^2^ = 95.4%.

*Gynostemma pentaphyllum* has shown significant potential in managing diabetes, particularly in reducing blood glucose levels (MD = −0.79 mmol/L, 95% CI = −1.08 to −0.51) (Figure 2A). The analysis demonstrated random-effects models support the hypoglycemic effect of *Gynostemma pentaphyllum*, with statistically significant results. However, there was moderate to high heterogeneity across the studies (*I* ^*2*^ = 66.1%). The treatment duration plays a crucial role, with a more consistent and significant effect observed in patients treated for more than 60 days (MD = −0.91 mmol/L, 95% CI = −1.14 to −0.68 vs. MD = −0.57 mmol/L, 95% CI = −0.65 to −0.24) (Supplementary Figure S3). While blood glucose reduction was evident across various studies, heterogeneity remained, and age did not significantly impact the outcomes. Patients under 50 years exhibited a more consistent effect, though not statistically significant (MD = −0.83 mmol/L, 95% CI = −1.25 to −0.41 vs. MD = −0.78 mmol/L, 95% CI = −1.17 to −0.39), while those over 50 showed more consistent reductions in blood glucose, albeit with higher heterogeneity (*I* ^*2*^ = 85% vs. *I* ^*2*^ = 0%) (Supplementary Figure S4). Subgroup analysis based on the preparation method revealed distinct variations in glycemic efficacy. Notably, the traditional decoction method yielded a more pronounced reduction in FPG (MD = −0.97 mmol/L, 95% CI = −1.56 to −0.37) compared to the administration of total glycosides or concentrated extracts (MD = −0.71 mmol/L, 95% CI = −1.02 to −0.41) (Supplementary Figure S5). Overall, *Gynostemma pentaphyllum* was an effective treatment for blood glucose control, particularly when used for extended periods and as a monotherapy.

*Gynostemma pentaphyllum* also demonstrated significant potential in reducing glycated hemoglobin (HbA1c) levels, confirming its efficacy as a treatment for diabetes (MD = −1.01%, 95% CI = −1.41 to −0.61) (Figure 2B). While subgroup analyses did not find significant differences between monotherapy and combination therapy (MD = −1.00%, 95% CI = −1.75 to −0.25 vs. MD = −1.01%, 95% CI = −1.41 to −0.61) (Supplementary Figure S6), the overall effect was statistically significant, indicating that *Gynostemma pentaphyllum* effectively lowers HbA1c. Despite the absence of a subgroup analysis based on treatment duration, the overall analysis supported its hypoglycemic efficacy. Based on the analysis of 2-h postprandial glucose (2hPG) levels, *Gynostemma pentaphyllum* demonstrated a clear hypoglycemic effect (*I* ^*2*^ = 95.4%), significantly reducing 2hPG. (MD = −0.90 mmol/L, 95% CI = −1.95 to −0.15) (Figure 2C).

The overall analysis indicated consistent benefits, supporting its effectiveness as a blood glucose-lowering agent. Subgroup analysis revealed that treatments lasting over 60 days produced more pronounced and consistent reductions in FPG, highlighting the importance of treatment duration.

Based on the analysis of insulin resistance, we conducted a preliminary evaluation of the effects of *Gynostemma pentaphyllum* on insulin resistance to lay the groundwork for further mechanistic studies (Supplementary Figure S7). Although the available clinical trials are limited, the herb showed potential in improving insulin sensitivity, suggesting it may help reduce insulin resistance. However, due to the small number of relevant clinical studies, further research is needed to better understand the underlying mechanisms and confirm its clinical efficacy.

## 3 Systematic literature review on the hypoglycemic mechanisms of *Gynostemma pentaphyllum*

*Gynostemma pentaphyllum* is a traditional herb that is often processed into tea and consumed as an aqueous infusion (Fan et al., 2017). It is believed to enhance health and was officially recognized by China’s National Health Commission in 2002 when it was included in the list of health supplements (Li, 2018; Shen et al., 2020; Wei et al., 2024). Scientific research has identified its main chemical constituents as saponins and polysaccharides, which impart a variety of pharmacological activities including anti-tumor, anti-inflammatory, antiviral, anti-aging properties, along with uric acid-lowering effects, lipid modulation, and protection of cardiovascular and liver health (Pang, 2016; Song et al., 2021; 2021; Wang et al., 2023; Lin et al., 2024).

*Gynostemma pentaphyllum* has shown multiple positive effects in the treatment of diabetes and its complications. Specifically, Gynostemma saponins can inhibit advanced glycation end-products (AGEs)-induced glomerular cell proliferation and extracellular matrix secretion, thus reducing glomerular injury (Tang et al., 2015). Additionally, it regulated the expression of vascular cell adhesion molecule-1(VCAM-1) and peroxisome proliferator-activated receptor alpha (PPAR-α), inhibiting glomerular cell apoptosis (Zhao et al., 2017), and modulated miR-125 to reduce inflammatory responses and immune dysfunction associated with diabetic nephropathy (Zhou et al., 2024).

In terms of liver health, *Gynostemma* regulated bile acid pathways to decrease hepatic lipid accumulation, promoting lipid and glucose metabolism (Wang, 2017). It also downregulated inflammatory factors and upregulates antioxidant enzyme activity, improving metabolic disorders in fatty liver disease (Zhao Q. et al., 2016). These actions helped alleviate symptoms of non-alcoholic fatty liver disease (NAFLD). Moreover, by regulating autophagy-related genes Beclin-1 and LC3B, *Gynostemma* improved glucose and lipid metabolism and reduced hepatic lipidosis (Zhang et al., 2018).

Regarding retinopathy, *Gynostemma* protected the retina by suppressing inflammatory reactions, reducing the occurrence of retinopathy (Lei, 2023). Further studies have shown that the GPA component in *Gynostemma* inhibits Keap1 to activate the Nrf2-dependent antioxidant pathway, reversing high-glucose-induced ferroptosis in human retinal microvascular endothelial cells (HRMECs), providing additional protective benefits (Wang et al., 2018; Zhuo et al., 2018; Shi, 2020; Chen et al., 2024).

**FIGURE 3:**
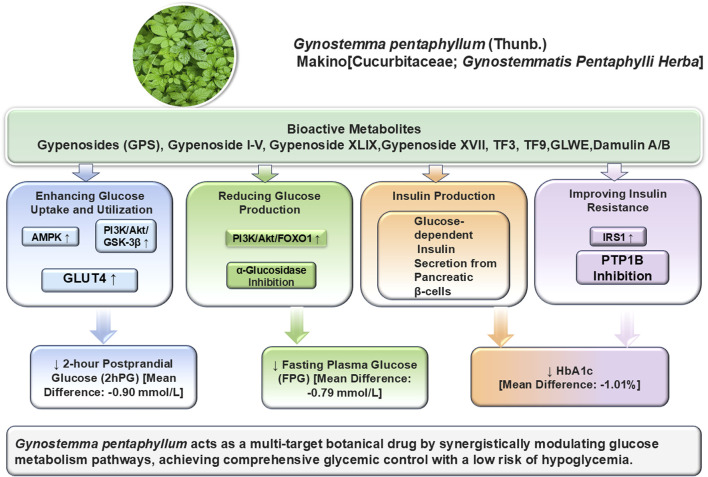
Bioactive metabolites and antidiabetic mechanisms of Gynostemma pentaphyllum. The schematic illustrates the multi-target synergistic effects of Gynostemma pentaphyllum on glucose metabolism. Bioactive metabolites (including Gypenosides, TF3, TF9, GLWE, and Damulin A/B) exert their effects through four primary pathways: enhancing glucose uptake and utilization (via AMPK, PI3K/Akt/GSK-3β, and GLUT4), reducing glucose production (via PI3K/Akt/FOXO1 and α-Glucosidase inhibition), promoting glucose-dependent insulin secretion from pancreatic β-cells, and improving insulin resistance (via IRS1 and PTP1B inhibition). These mechanisms collectively lead to significant reductions in 2-hour postprandial glucose (2hPG), fasting plasma glucose (FPG), and glycated hemoglobin (HbA1c), achieving comprehensive glycemic control with a low risk of hypoglycemia.

*Gynostemma pentaphyllum* has demonstrated considerable potential in managing diabetes through multiple mechanisms, including reducing glucose production, enhancing glucose uptake, and improving insulin sensitivity (Lin and Chen, 2011; Zhao Q. et al., 2016). Furthermore, these effects suggested that *Gynostemma* could be a promising natural treatment for diabetes, offering a multifaceted approach to improving glucose control and insulin function (Figure 3; Table 2).

**TABLE 2:** Studies on the compounds in Gynostemma pentaphyllum for diabetes treatment.

Component	Experimental model	Mechanism	Effect	Author	Publication year
Gynostemma saponin XVII	Enzyme activity test, Caco-2 cells	Regulates PI3K/Akt/FOXO1 signaling pathway, improves FOXO1 phosphorylation level, inhibits overexpression of PEPCK and G6Pase	Inhibits hepatic gluconeogenesis, reduces glucose production	Zhao, M., et al.	2023
Gynostemma endophytic fungus polysaccharide (JY25 polysaccharide)	Enzyme activity test, Caco-2 cells	Inhibits α-glucosidase	Reduces glucose absorption	Zhao, X. X., et al.	2016
HPFr:C14	HepG2 cell	The compounds contained inhibit α-glucosidase	Reduces glucose absorption	Li, Y., et al.	2018
Gynostemma leaf water extract	Streptozotocin (STZ)-induced diabetic rats	Increases GLUT-4 expression in the gastrocnemius muscle of diabetic rats	Enhances glucose uptake by muscle cells	Wang, T. Z., et al.	2020
Gynostemma total saponins (GPS)	Rats with type 2 diabetes mellitus	Enhances insulin signaling pathway	Promotes glycogen synthesis, increases glucose utilization, improves insulin sensitivity	Zhu, K. N., et al.	2021
DamulinA, DamulinB	L6 myotube cells	Activates AMPK pathway	Increases fatty acid oxidation and glucose uptake, enhances glucose uptake	P. H. Nguyen, et al.; R. Gauhar, et al.	2010; 2011
10 new dammarane-type triterpenoid compounds	Differentiated 3T3-L1 adipocyte cells; differentiated mouse C2C12 skeletal myoblasts	Activates AMPK pathway	Promotes glucose uptake	H. T. T. Pham, et al.	2018
Gynostemma total saponins	Diabetic rats	Not mentioned	Reduces blood glucose, blood lipids, and free fatty acids in diabetic rats, enhances insulin levels, improves insulin sensitivity, reduces insulin resistance index	Pang, Y. P., et al.	2016
TF9, TF3, compounds 20A, 20B	Male diabetic GK rats; pancreatic islet cells	TF9 enhances insulin release at both high and low glucose concentrations, TF3 significantly increases insulin secretion at high glucose concentrations, compounds 20A and 20B stimulate insulin secretion at high glucose concentrations	Regulates insulin secretion, reduces hypoglycemia risk	Lena C. E. Lundqvist, et al.	2019
Gynostemma saponin XLIX (Gyp-XLIX)	Fat emulsion infusion model rats	Inhibits abnormal phosphorylation of IRS1 and enhances PI3K/Akt signaling pathway activation	Increases steady-state glucose infusion rate, alleviates insulin resistance, enhances insulin sensitivity	Shi, S. L., et al.	2020
Compounds 5a–5c, compounds 1, 3, 12, 13, 14, gypensapogenin III	Protein tyrosine phosphatase 1B (PTP1B); T-cell protein tyrosine phosphatase (TCPTP)	PTP1B inhibition activity	Inhibits PTP1B, improves insulin sensitivity, alleviates diabetes symptoms	Ji-Qing Xu, et al.; Xiao-Shu Zhang, et al.; Daopeng Tan, et al.	2010 2013 2023

### 3.1 Reducing glucose production

Gluconeogenesis is the primary source of glucose in the liver, with phosphoenolpyruvate carboxykinase (PEPCK) and glucose-6-phosphatase (G6Pase) being key regulatory enzymes (Petersen et al., 2017). FOXO1 promotes gluconeogenesis by activating the expression of PEPCK and G6Pase. Zhao et al.'s research (Zhao, 2023) revealed that Gynostemma saponin XVII (Supplementary Figure S8A) significantly inhibited glucose production in the liver by regulating the PI3K/Akt/FOXO1 signaling pathway. By enhancing the phosphorylation level of FOXO1, Gynostemma saponin XVII effectively suppressed the overexpression of PEPCK and G6Pase, thereby reducing the process of gluconeogenesis and glucose production. Zhao et al.'s study (Zhao X. et al., 2016) delved into the inhibitory effect of JY25 polysaccharide, a polymer composed of more than 10 monosaccharide molecules linked by glycosidic bonds, derived from endophytic fungi in *Gynostemma*, on α-glucosidase. Under optimal experimental conditions, JY25 polysaccharide demonstrated significant inhibitory effects, with inhibition rates increasing from 7.37% to 62.44%, reaching a maximum of 68.21% at a concentration of 6.15 mg/mL. Although its inhibitory activity was slightly weaker than that of acarbose, in the Caco-2 cell model, the mechanism of inhibition by JY25 polysaccharide was similar to acarbose, indicating its potential in reducing glucose absorption. Li (2018) further explored the chemical constituents and α-glucosidase inhibitory activity of the HPFr.C14 fraction from Gynostemma, identifying four major compounds: Vincetoxicoside A (Supplementary Figure S8B), rutin (Supplementary Figure S8C), kaempferol-3-O-glucose-7-O-rhamnoside (Supplementary Figure S8D), and Nicotiflorine (Supplementary Figure S8F). These compounds exhibited α-glucosidase inhibitory activity. Wang et al. (2012) showed that rutin has a better inhibitory effect on α-glucosidase than the commonly used drug acarbose, demonstrating its potential in aiding blood glucose reduction. Wu (2010) suggested that rutin’s enzyme inhibitory effect might be related to its 3’,4’-dihydroxyphenyl group structure.

These research findings collectively revealed the mechanisms by which various compounds from *Gynostemma* reduce glucose production in the body, highlighting *Gynostemm*a in inhibiting glucose production.

### 3.2 Enhancing glucose uptake and utilization

Wang et al. (2020) indicated that Gynostemma leaf water extract enhanced glucose uptake in muscle cells by increasing the expression of GLUT-4 in the gastrocnemius muscle of diabetic rats. Although no significant changes were observed at the mRNA and total protein levels, immunofluorescence results showed that Gynostemma leaf water extract could reverse the decrease in GLUT-4 expression in the diabetic model group, promoting glucose uptake. Additionally, Zhu et al. (2021) found that the relative expression level of GLUT4 mRNA in the skeletal muscle of the model group decreased, while the relative expression level of NF-κB mRNA in the adipose tissue increased. The high-dose Gynostemma total saponins group could reverse this effect. Zhao (2023) demonstrated that after treating insulin-resistant HepG2 cells with Gynostemma saponin XVII (Supplementary Figure S8A) for 24 h, their glucose uptake ability gradually improved. These results suggested that Gynostemma saponin XVII can effectively improve glucose uptake in the HepG2 cell IR model within a concentration range of 0.1–10 μM, with the improvement being concentration-dependent. Studies (Gao et al., 2019; Qi et al., 2022; Malik et al., 2023) have shown that the PI3K/Akt/GSK-3β signaling pathway was considered a crucial signal transduction pathway, playing a key role in regulating intracellular glucose uptake and glycogen synthesis. After insulin binds to its receptor, it activated PI3K and its downstream Akt, further inhibiting the activity of GSK-3β, thereby promoting GLUT4 translocation and glycogen synthesis. Subsequent research (Shi, 2020) has found that Gynostemma saponin XVII improves insulin signaling by regulating the PI3K/Akt/GSK-3β pathway in IR-HepG2 cells, promoting glycogen synthesis, and increasing glucose utilization. Nguyen’s study (Nguyen et al., 2011) isolated two new dammarane saponins (Damulin A, Damulin B) (Supplementary Figure S8F; Supplementary Figure S8G) from Gynostemma leaves, which increased fatty acid oxidation and glucose uptake by activating the AMPK pathway. R. Gauhar’s research (Gauhar et al., 2012) further confirmed that the Gynostemma extract’s Damulin A and Damulin B are potent AMPK activators, enhancing glucose uptake by increasing GLUT4 translocation. The content of damulin A and B has increased with the increase of extraction time. According to Pham et al. (2018), 10 new dammarane triterpenoids isolated from a 70% ethanol extract of Gynostemma also activated the AMPK pathway, compounds 1, 2, and 4 (Supplementary Figure S8H; Supplementary Figure S8I; Supplementary Figure S8G) markedly increased glucose uptake and promoted the translocation of GLUT4 by modulating the AMPK signaling pathway.

These findings collectively revealed the diverse mechanisms by which various compounds in *Gynostemma* enhanced glucose uptake and utilization.

### 3.3 Insulin production

Pang (2016) showed that Gynostemma total saponins reduced blood glucose, lipid, and free fatty acid levels in diabetic rat models while increasing insulin levels and sensitivity, reducing the insulin resistance index. These results indicated that Gynostemma total saponins not only directly lowered blood glucose but also alleviated diabetic symptoms by enhancing insulin production and improving insulin sensitivity. Lundqvist’s study (Lundqvist et al., 2019) used HPLC to separate and analyze Gynostemma extracts, finding that compound TF9 enhances insulin release at both low and high glucose concentrations, while TF3 exhibits dose-dependent effects, significantly promoting insulin secretion at high glucose concentrations but not at low glucose concentration. Further studies also found that 20A and 20B were main compounds of TF3, which stimulated insulin secretion at high glucose concentrations, with 20B (gylongiposide I) (Supplementary Figure S8K) only being effective at high glucose concentrations.

These findings revealed the specific mechanisms of Gynostemma compounds in regulating insulin secretion, reducing the risk of hypoglycemia and providing a basis for treatment of diabetes.

### 3.4 Insulin resistance

Zhu et al. (2021) showed that Gynostemma total saponins (GPS) reduced the insulin resistance index (HOMA-IR) in diabetic rats at high doses, effectively alleviating insulin resistance. Additionally, the insulin sensitivity index (ISI) significantly increased in the high-dose GPS group and all treatment groups, further confirming the potential of GPS in enhancing insulin sensitivity and reducing insulin resistance. Shi’s study (Shi, 2020) using a lipid emulsion infusion model, demonstrated that Gynostemma saponin XLIX improved the steady-state glucose infusion rate (SSGIR), thereby alleviating lipid emulsion-induced insulin resistance. Its mechanism involved inhibiting the abnormal phosphorylation of IRS1, enhancing the activation of the PI3K/Akt signaling pathway, improving insulin signaling transmission, increasing insulin sensitivity, and reducing the occurrence of insulin resistance.

Protein tyrosine phosphatase 1B (PTP1B) is a key enzyme that negatively regulated insulin signaling by dephosphorylating insulin receptors and downstream signaling molecules, thereby weakening insulin signaling. Overexpression or enhanced activity of PTP1B was one of the important factors leading to insulin resistance in type 2 diabetes. Inhibiting PTP1B activity can enhance insulin signaling, improve insulin sensitivity, alleviate insulin resistance, and lower blood glucose levels (Elchebly et al., 1999; Rondinone et al., 2002; Koren and Fantus, 2007). Yao et al. (2023) demonstrated that compound XLIX (Supplementary Figure S8A), derived from Gynostemma, exhibited significant potential in alleviating insulin resistance by enhancing the phosphorylation of PPP1CB at Thr316, which was crucial for regulating insulin signaling. Treatment with XLIX not only improved lipid profiles and reduced liver fat accumulation but also notably enhanced insulin sensitivity in both HFD-induced NAFLD and HFHC diet-induced NASH models. Furthermore, Xu’ work (Xu et al., 2010) led to the discovery of several compounds with notable PTP1B inhibitory activity. Among these, compound 5b (Supplementary Figure S8N) exhibited the highest potency, being 42 times more effective than the original compound 2b (Supplementary Figure S8L).

Compound 2b was a naturally derived sapogenin obtained via the acid hydrolysis of 1b, an abundant natural triterpene saponin found in *Gynostemma pentaphyllum*. This process removed the sugar moiety from the natural precursor 1b, yielding 2b, which inherently demonstrated moderate PTP1B inhibitory activity. The discovery of this natural biological activity highlights the intrinsic value of *Gynostemma pentaphyllum*. Furthermore, the natural dammarane scaffold of 2b has proven to be an excellent template for drug discovery. Zhang et al. (2013) further isolated compounds with PTP1B inhibitory activity, namely, compounds1, 3, 12, 13, and 14. The study indicated that the biological activity of dammarane-type triterpenoid compounds is associated with the hydroxyl group at the C-3 position and the configuration at the C-23 position. In Tan’s research (Tan et al., 2023), five new dammarane-type triterpenoid compounds have been isolated and named Gypensapogenin I to V. Notably, Gypensapogenin I (Supplementary Figure S8O) possessed an unprecedented skeletal structure. Through molecular docking simulations, the binding modes of these triterpenoids with the PTP1B protein were predicted. The results showed that all compounds exhibited good affinity with PTP1B. Particularly, Gypensapogenin III (Supplementary Figure S8P) formed competitive hydrogen bonds between its side-chain hydroxyl group and the Asp48 amino acid residue of PTP1B, which may be a critical mechanism for its PTP1B inhibitory activity.

The various active components in Gynostemma exhibit potential in alleviating type 2 diabetes and related metabolic disorders by improving insulin signaling and sensitivity through PTP1B inhibition, providing an important basis for the development of new natural drugs.

## 4 Discussion

### 4.1 Clinical efficacy and the significance of treatment duration

The meta-analysis results demonstrate that *Gynostemma pentaphyllum* improved glycemic control, evidenced by the reductions in FPG, 2hPG, and HbA1c (Figure 2; Figure 3). Our subgroup analysis reveals that treatment duration is a critical factor, with more pronounced benefits observed in interventions lasting over 60 days (Supplementary Figure S3). This suggested that the specialized metabolites in this botanical drug may require a cumulative period to achieve stable metabolic reprogramming, particularly for long-term markers like HbA1c, which reflects the average blood glucose levels over the preceding 2–3 months.

### 4.2 Translational perspective: bridging molecular mechanisms to clinical outcomes

As visually synthesized in our redesigned Figure 3, the clinical outcomes are direct macroscopic reflections of the coordinated pharmacological actions exerted by *Gynostemma pentaphyllum* metabolites. Specifically, the significant reduction in FPG is primarily driven by the botanical drug’s ability to suppress hepatic glucose overproduction. This process is mediated by the regulation of gluconeogenic enzymes such as PEPCK and G6Pase, which are controlled through signaling cascades like the PI3K/Akt pathway (Song et al., 2022; Lee et al., 2025). Concurrently, the improvement in 2hPG reflects an enhanced efficiency in peripheral glucose clearance. Bioactive components facilitate the rapid recruitment of GLUT4 transporters to the cell membrane by activating metabolic sensors like AMPK, ensuring that postprandial glucose is effectively utilized by skeletal muscles rather than remaining elevated in circulation (Song et al., 2022).

Furthermore, the long-term stabilization of HbA1c is fundamentally supported by the systematic amelioration of insulin resistance. Our analysis of core triterpenoid scaffolds confirms that these substrates, by modulating targets such as PTP1B, restore cellular insulin signaling sensitivity (Xie et al., 2024). This provided a robust biological foundation for the sustained glycemic control observed across the included clinical trials. By explicitly linking these molecular events to specific clinical endpoints, we transform the detailed mechanistic data into a cohesive translational narrative that explains the botanical drug’s therapeutic efficacy (Wang et al., 2019).

### 4.3 Impact of preparation diversity and phytochemical standardization

A key strength of this review is the explicit distinction between various forms of the botanical drug (Table 3). While whole-plant preparations like botanical teas provide a synergistic “entourage effect” of polysaccharides and flavonoids to modulate α-glucosidase activity, concentrated extracts such as Gypenosides offer a more potent, targeted intervention on intracellular insulin signaling (Thupakula et al., 2024). Our retrospective assessment using the ConPhyMP guidelines underscores a critical reporting gap in the primary literature regarding phytochemical fingerprinting and the quantification of marker compounds, which hinders the reproducibility of clinical efficacy (Heinrich et al., 2026).

**TABLE 3:** Characteristics of Gynostemma pentaphyllum preparations in the included clinical studies.

Study	Preparation type	Plant part used	Extraction solvent and method	Standardized marker content
Chen et al. (2023)	Total glycosides tablets	Tender leaves and buds (noted in intro)	Commercial preparation	Total glycosides (20 mg per tablet)
Ma et al. (2017)	Water infusion/Tea (5 g/dose)	Tender leaves and buds	Steeped in boiling water	Unstandardized raw herb
Yu (2017)	Total glycosides tablets	Not explicitly specified	Commercial preparation	Total glycosides (quantity not detailed)
Shi and Fang (2016)	Commercial tablet (0.5 g/dose)	Not explicitly specified	Commercial preparation	Not specified
Zhang et al. (2015)	Traditional decoction (30 g/day)	Not explicitly specified	Water decoction	Not specified
Huyen et al. (2012)	Soluble extract powder, 6 g/day	Whole plants	Boiling water extraction for 2h, precipitation of impurities using 70% ethanol	Verified for flavonoids and saponins; contains approx. 18% saponins
Huyen et al. (2010)	Soluble extract powder (administered as tea), 6 g/day	Whole plants	Boiling water extraction for 2h, precipitation of impurities using 70% ethanol	Verified for flavonoids and saponins; contains approx. 18% saponins
Wang et al. (1998)	Water infusion/Tea (10 g/dose)	Wild herb	Steeped as tea	Not specified

### 4.4 Safety, tolerability, and medicine food homology status

Consistent with its status as a “medicine food homology” item—functioning as both a functional food and a nutraceutical—*Gynostemma pentaphyllum* was generally well-tolerated across the reviewed trials. Our systematic review of safety data reveals that most included studies reported no severe adverse events or severe hypoglycemia. Minor gastrointestinal symptoms were infrequent and transient, affirming the safety profile of this botanical drug for general glycemic management (Wang et al., 1998; Huyen et al., 2010; 2012; Zhang et al., 2015; Shi and Fang, 2016; Yu, 2017; Shi, 2020; Chen et al., 2023). However, the current lack of stratified pharmacokinetic data and biomarker-driven patient selection prevents the classification of these effects as “precision treatment.” Instead, the evidence suggests a robust, general hypoglycemic benefit that is suited for integrative or preventive care (Giné-Balcells et al., 2026; Ma et al., 2026).

### 4.5 Limitations and future perspectives

Despite the positive findings, this study has limitations. The primary clinical trials were often small-scale and lacked rigorous phytochemical standardization according to modern pharmacopeial monographs. Looking forward, we propose that *Gynostemma pentaphyllum* represents a paradigm for “food-derived prevention,” but its transition to a first-line therapeutic agent requires standardized extracts with defined metabolite profiles (Lee et al., 2025). Future research should prioritize large-scale, multi-center RCTs and utilize “omics” technologies to further elucidate the interaction between *Gynostemma pentaphyllum* metabolites and the gut microbiota, which may play a hidden role in its metabolic benefits (Guo et al., 2024). Additionally, rigorous investigation into the bioavailability and metabolic fate of specific Gypenosides in human subjects is essential to optimize dosage regimens.

In conclusion, this combined meta-analysis and systematic review provides compelling evidence for the efficacy of *Gynostemma pentaphyllum* in glycemic control. By bridging macroscopic clinical data with microscopic molecular pathways, we have elucidated how its diverse metabolites synergistically target hepatic glucose production, peripheral uptake, and insulin resistance. As a safe and effective nutraceutical, *Gynostemma pentaphyllum* serves as a valuable integrative option for diabetes management. However, its full clinical potential can only be realized through improved phytochemical quality control, standardized reporting, and a deeper understanding of its translational pharmacology.

## Data Availability

The original contributions presented in the study are included in the article/Supplementary Material, further inquiries can be directed to the corresponding authors.
