# Supplementary material for: Efficacy and mechanisms of Gynostemma pentaphyllum as a medicine food homology herb in glycemic control: a meta-analysis with review
Source: Front Pharmacol. 2026 Apr 15;17:1731723. doi: 10.3389/fphar.2026.1731723 (PMC13125874; doi:10.3389/fphar.2026.1731723)
Supplement: Supplementary file 1 [file Supplementaryfile1.docx]

**Supplementary Figure**


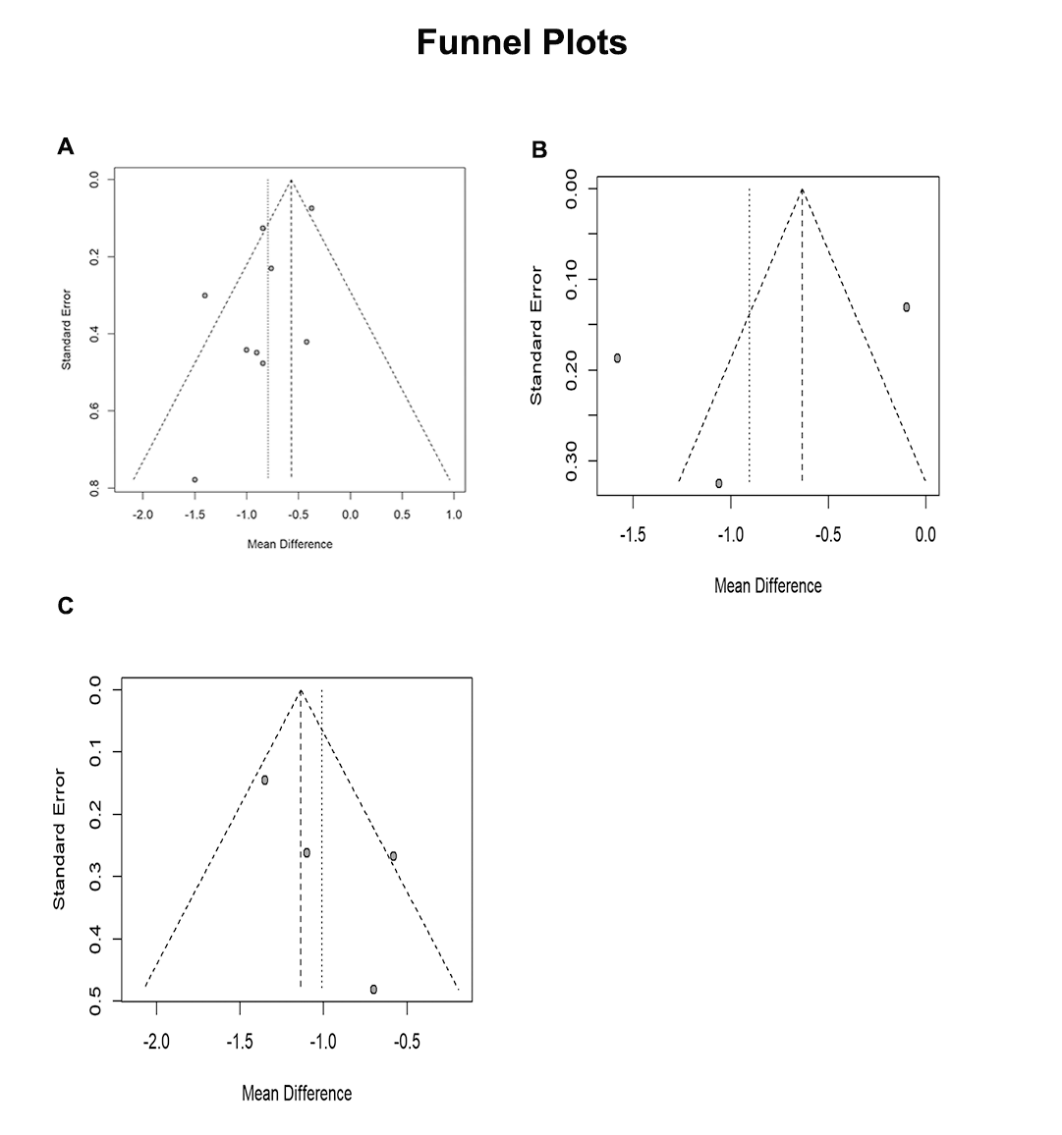


**Supplementary Figure 1. Funnel plots for FPG, HbA1c, and 2hPG in the meta-analysis.**

**A**. Funnel plot for the mean difference in Fasting Plasma Glucose (FPG). The plot showed a symmetric distribution, indicating the absence of publication bias. **B**. Funnel plot for the mean difference in HbA1c. The distribution was mostly symmetric, suggesting minimal bias. **C**. Funnel plot for the mean difference in 2-hour post-glucose (2hPG) values. The plot suggested a symmetrical distribution, reinforcing the reliability of the results.


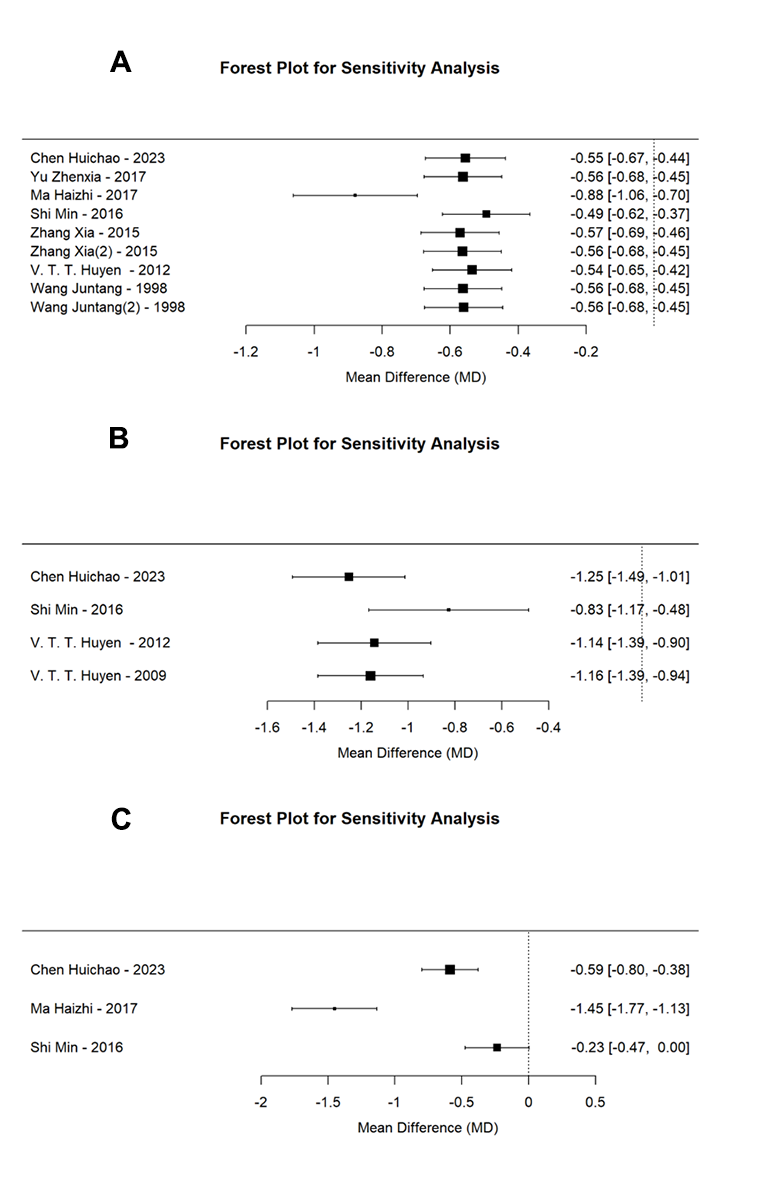


**Supplementary Figure 2. Forest plots for sensitivity analysis in the meta-analysis.**

**A**. Forest plot showing the mean differences (MD) for various studies included in the sensitivity analysis for fasting plasma glucose (FPG). The results of each study were displayed with their corresponding 95% confidence intervals. The studies were generally consistent, with mean differences ranging from -0.88 to -0.49. **B**. Forest plot for sensitivity analysis with studies from 2012 to 2023, showing a mean difference (MD) between -1.25 and 0.83. **C**. Forest plot for sensitivity analysis including studies from 2016 to 2023. This panel highlighted a wider range of mean differences, from -1.45 to -0.23, indicating variability in the results, particularly for the Ma Haizhi (2017) study.


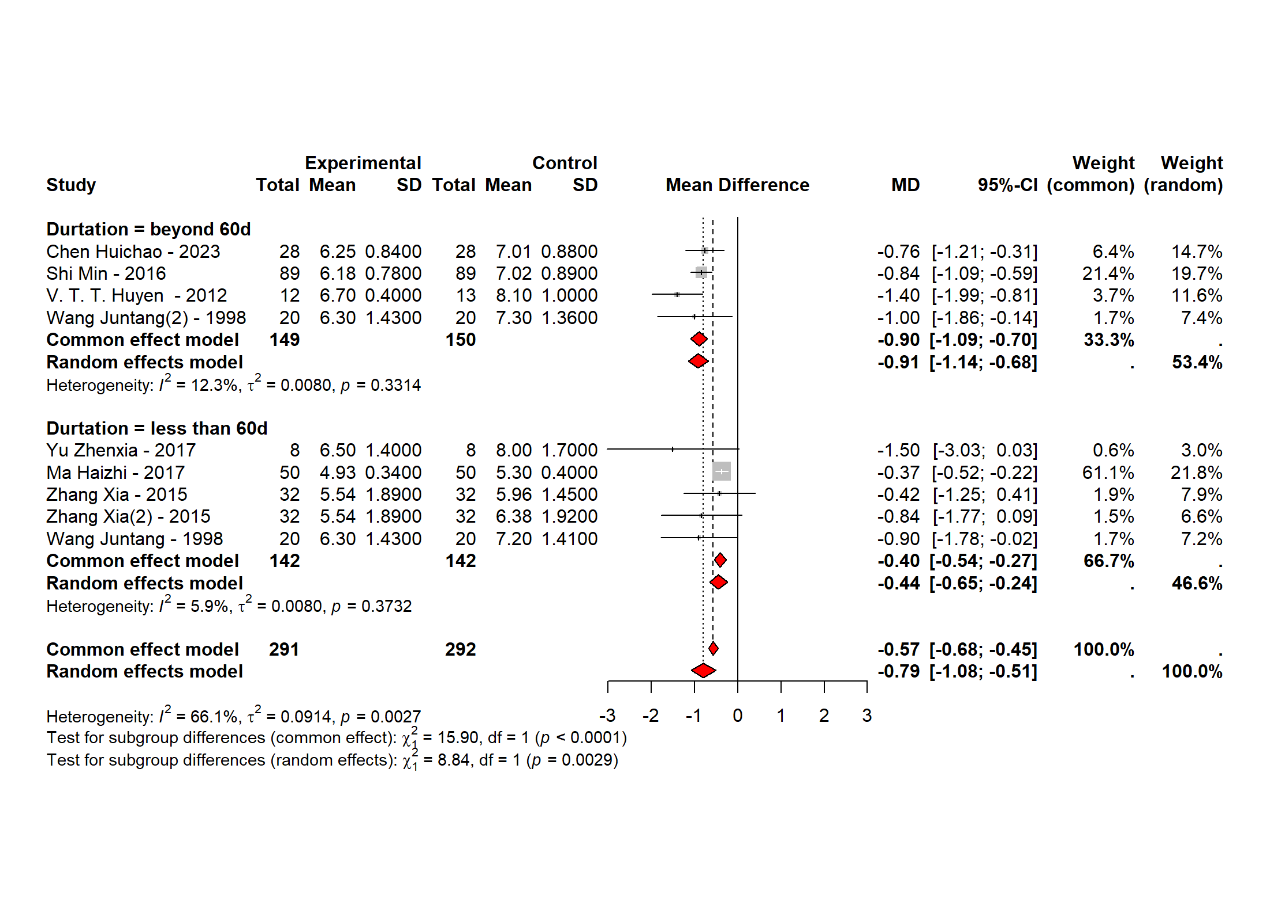


**Supplementary Figure 3. Subgroup analysis by treatment duration for Gynostemma pentaphyllum in blood glucose management.** Forest plot showing the mean differences (MD) for studies with a treatment duration of more than 60 days and less than 60 days.


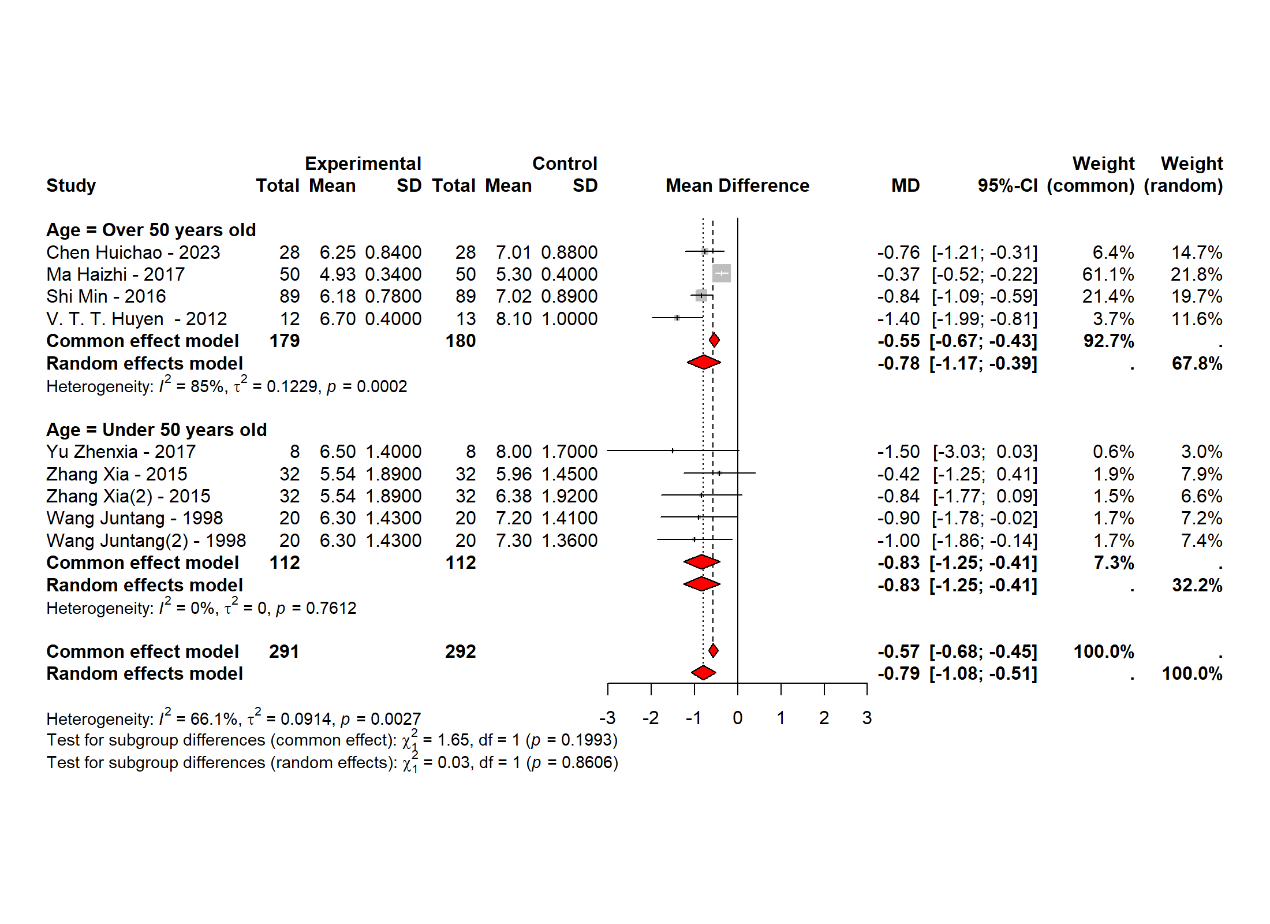
**Supplementary Figure 4. Subgroup analysis by age for Gynostemma pentaphyllum in blood glucose management.** Forest plot showing the mean differences (MD) for studies with participants over 50 years old. and under 50 years old.


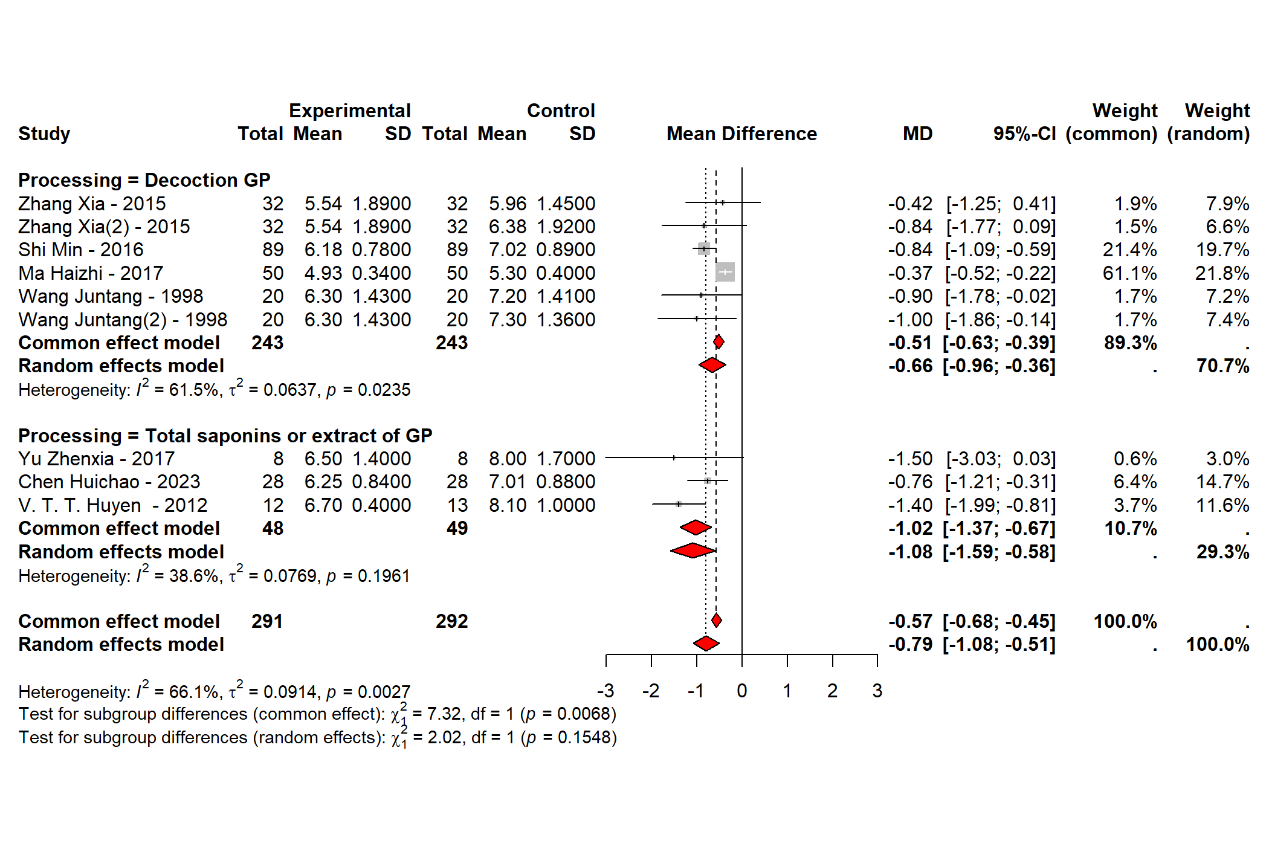


**Supplementary Figure 5. Subgroup analysis by processing method for *Gynostemma pentaphyllum* in blood glucose management.** Forest plot showing the mean differences (MD) in blood glucose levels, comparing two processing subgroups: Decoction GP (Gynostemma pentaphyllum) and Total saponins or extract of GP.


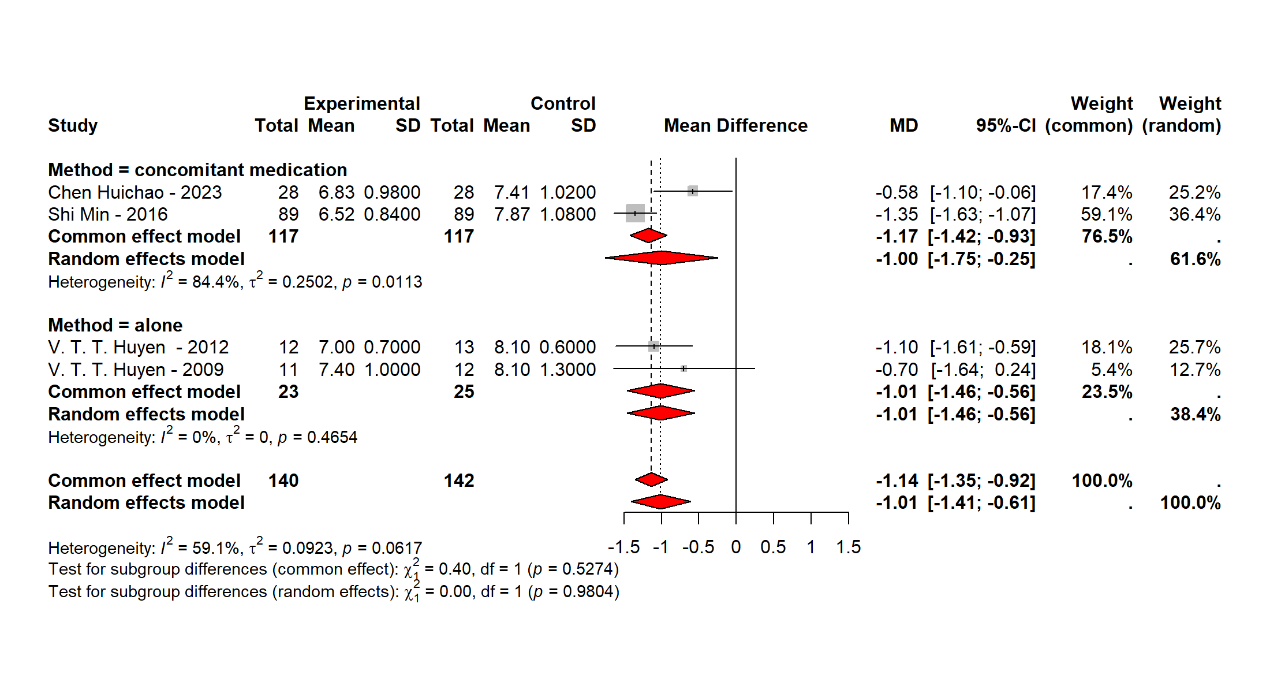


**Supplementary Figure 6. Subgroup analysis by intervention (Glycated Hemoglobin).** Forest plot showing the mean differences (MD) for studies where Gynostemma pentaphyllum was used in combination with other medications and alone.


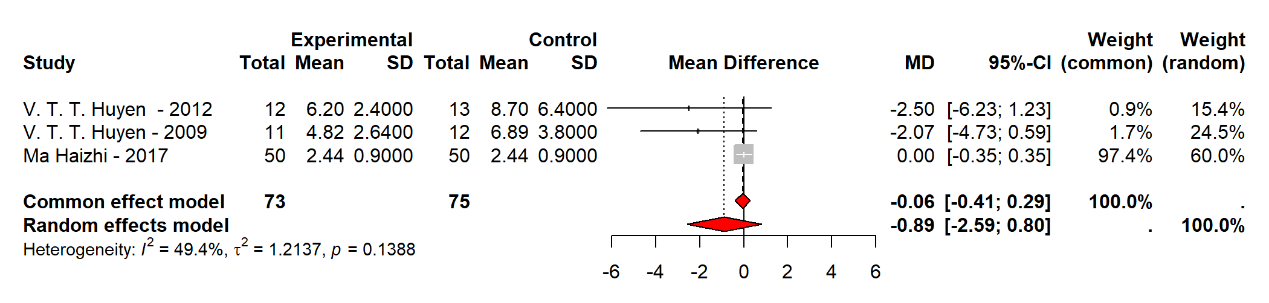


**Supplementary Figure 7. Homeostasis Model Assessment of Insulin Resistance (HOMA-IR).** This forest plot displayed the mean differences (MD) in HOMA-IR between experim


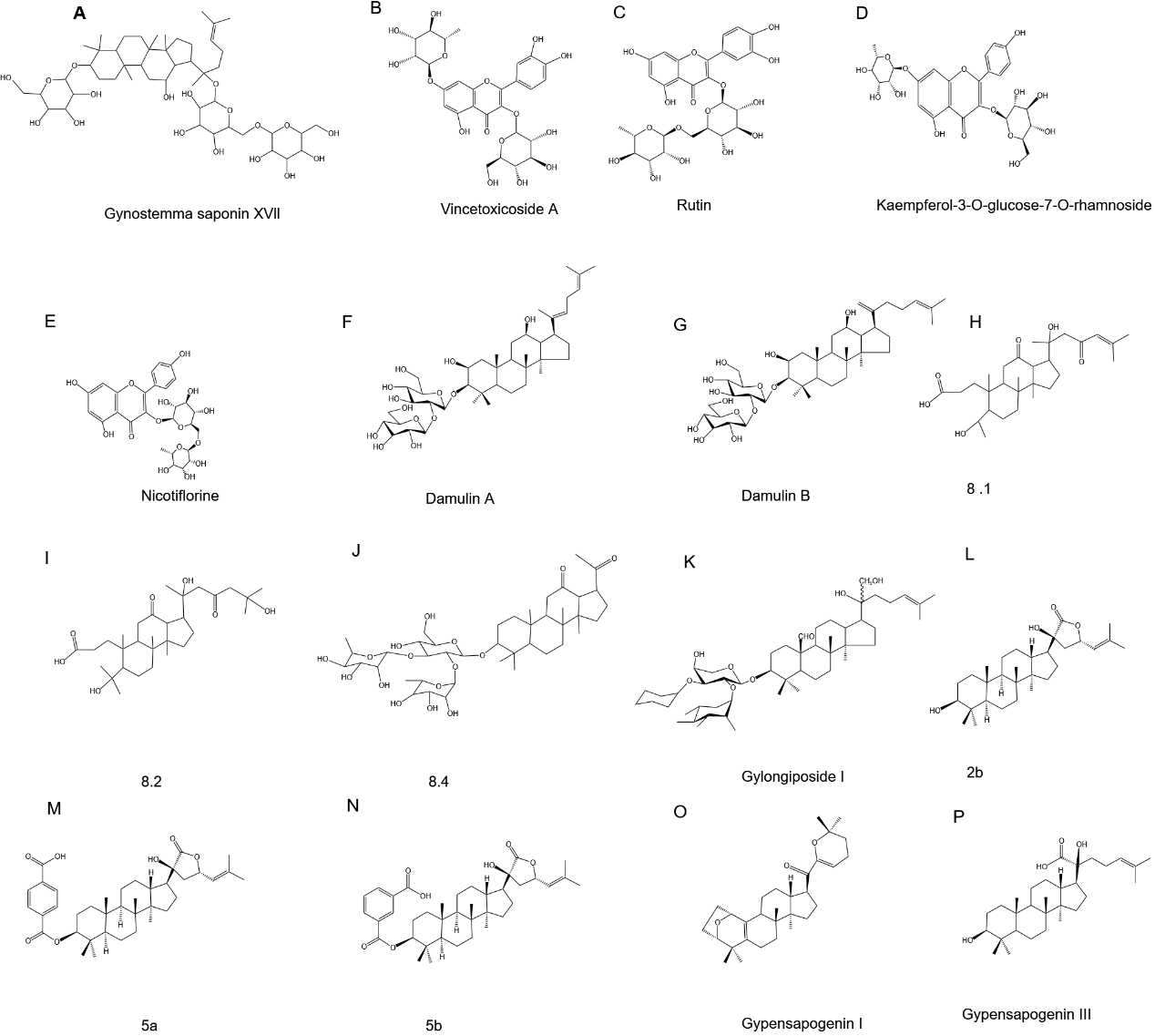


**Supplementary Figure 8.** The various active components shown in the figure all originate from Gynostemma pentaphyllum. These components exhibited significant anti-diabetic potential through different mechanisms and targets. Gynostemma pentaphyllum is rich in various bioactive substances.

ental and control groups across various studies.
